# Supplementary figures and images for: Weight trajectories in aging humanized APOE mice with translational validity to human Alzheimer’s risk population: A retrospective analysis
Source: PLoS One. 2025 Jan 24;20(1):e0314097. doi: 10.1371/journal.pone.0314097 (PMC11760569; doi:10.1371/journal.pone.0314097)

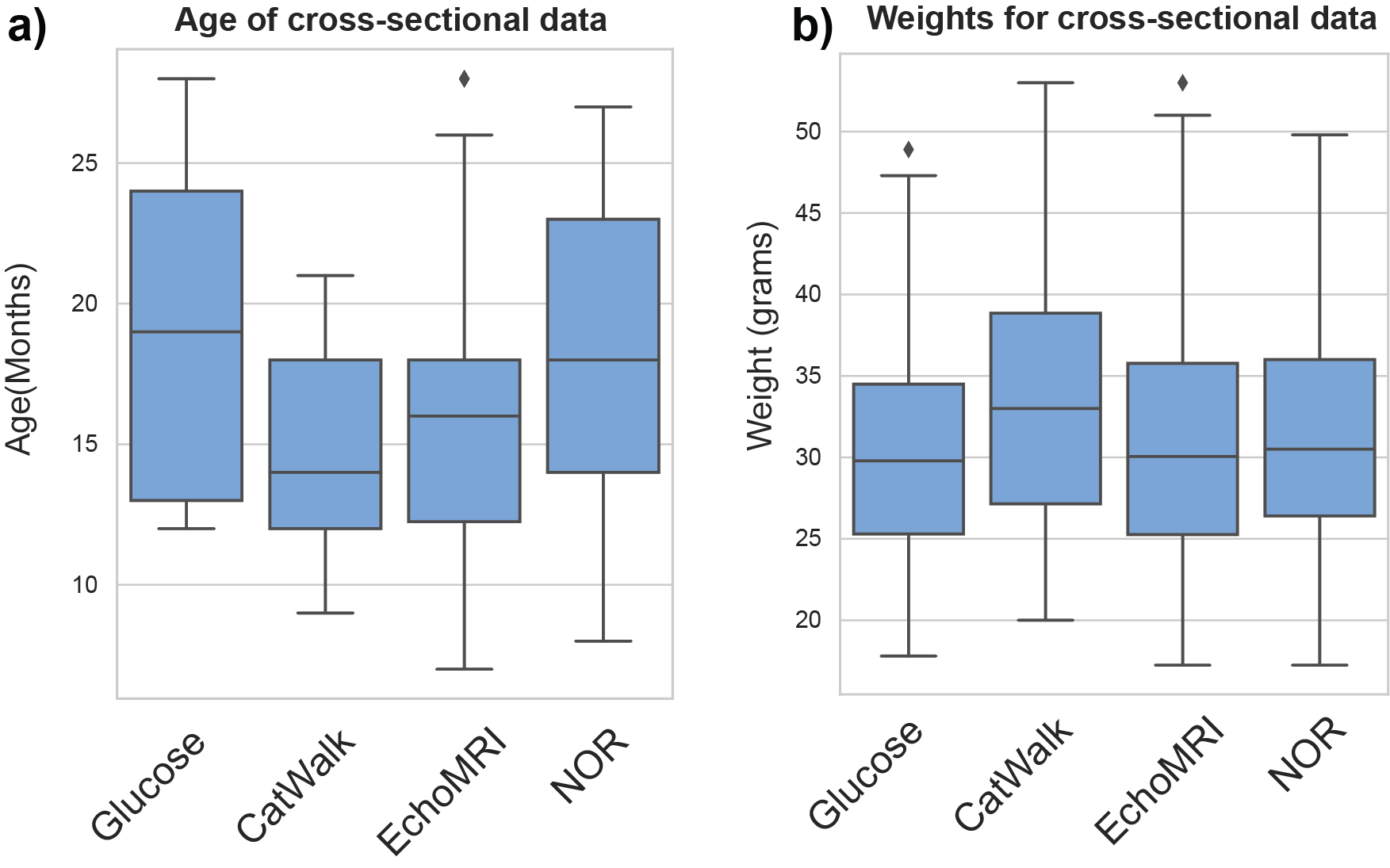

Supplement: S1 Fig — a) shows distribution of age for blood glucose, CatWalk, EchoMRI, and Novel Object Recognition (NOR), while b shows the distribution of mice weights in grams at the time of cross-sectional data collection. These distributions highlight the variability in age and weights of mice for which physiological and behavioral data were available. Cross-sectional data available for a small subset of mice: (i) 132 fasting blood glucose, (ii) 131 CatWalk™ XT, (iii) 298 EchoMRI™, and (iv) 133 NOR observations. Cross-sectional data are reported in S3 Table. (TIF) [file pone.0314097.s004.tif]

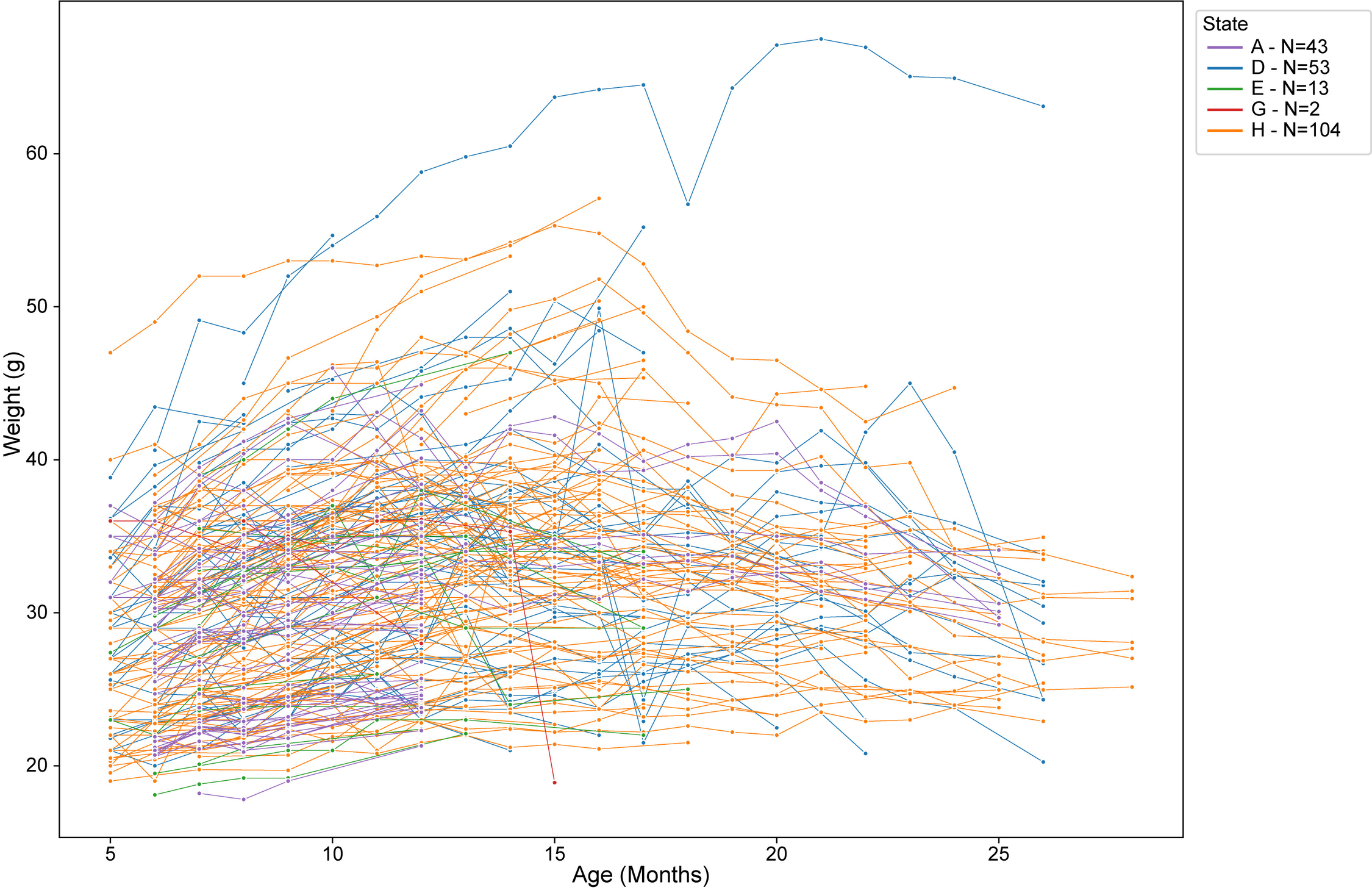

Supplement: S2 Fig — Lines are colored according to the last not stable hidden state (A, D, E, G, or H) associated to the mouse assigned by the Autoregressive Hidden Markov Model (AHMM). (TIF) [file pone.0314097.s005.tif]

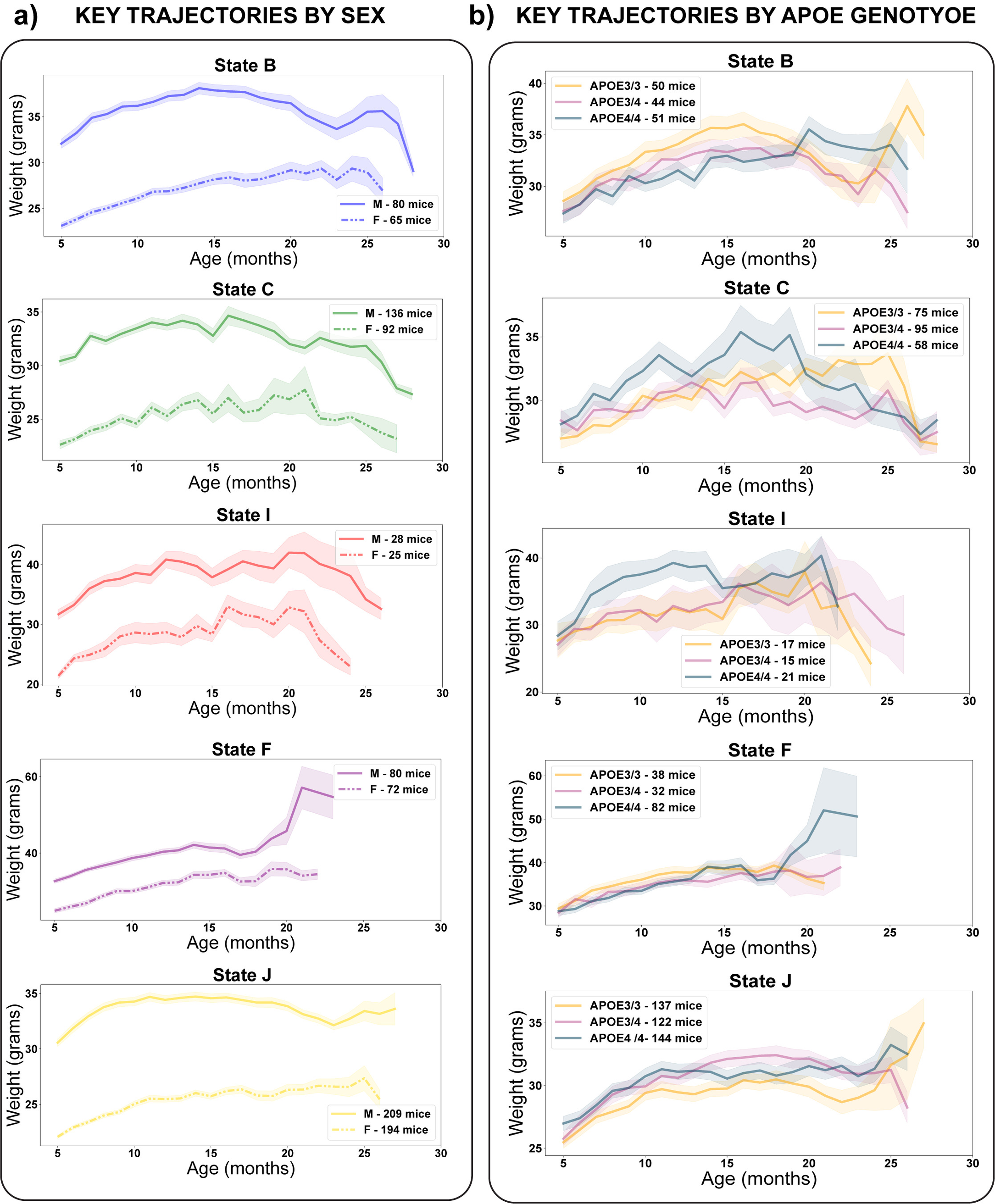

Supplement: S3 Fig — a) shows the 5 key weight trajectories (B, C, I, F and J) derived using Autoregressive Hidden Markov Model (AHMM) split by sex. b) shows the AHMM identified trajectories by APOE genotype. Legends report the number of mice belonging to each curve (subgroup). (TIF) [file pone.0314097.s006.tif]

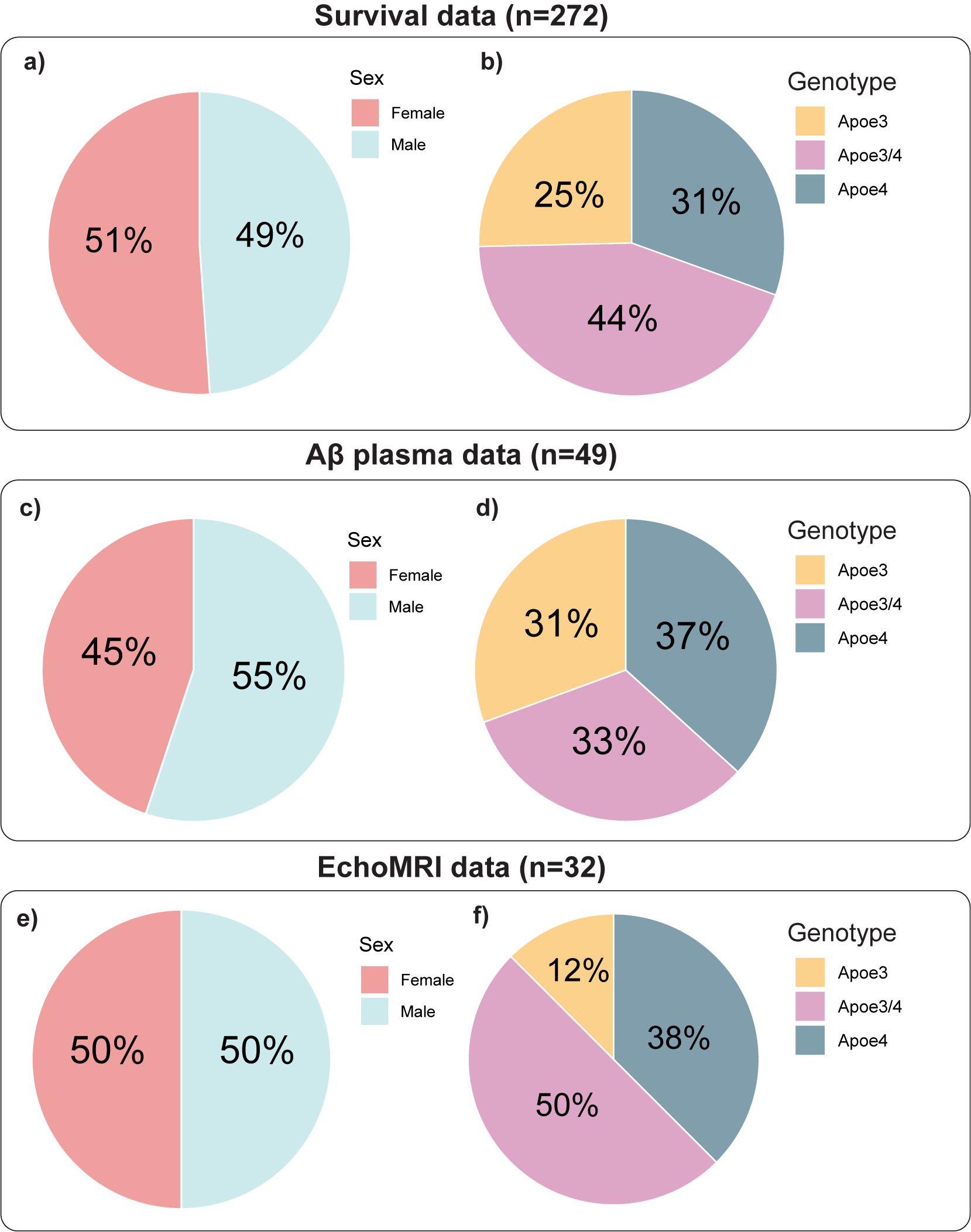

Supplement: S4 Fig — Percent of sex and APOE genotype distributions of survival data (panels a and b), Aβ plasma data (panels c and d), and EchoMRI™data (panels e and f). Panel a, c, and e show survival, Aβ plasma, and EchoMRI™ based on sex. Panels b, d, and f e show survival, Aβ plasma, and EchoMRI™ based on genotype. N indicates the number of mice in each dataset. (TIF) [file pone.0314097.s007.tif]

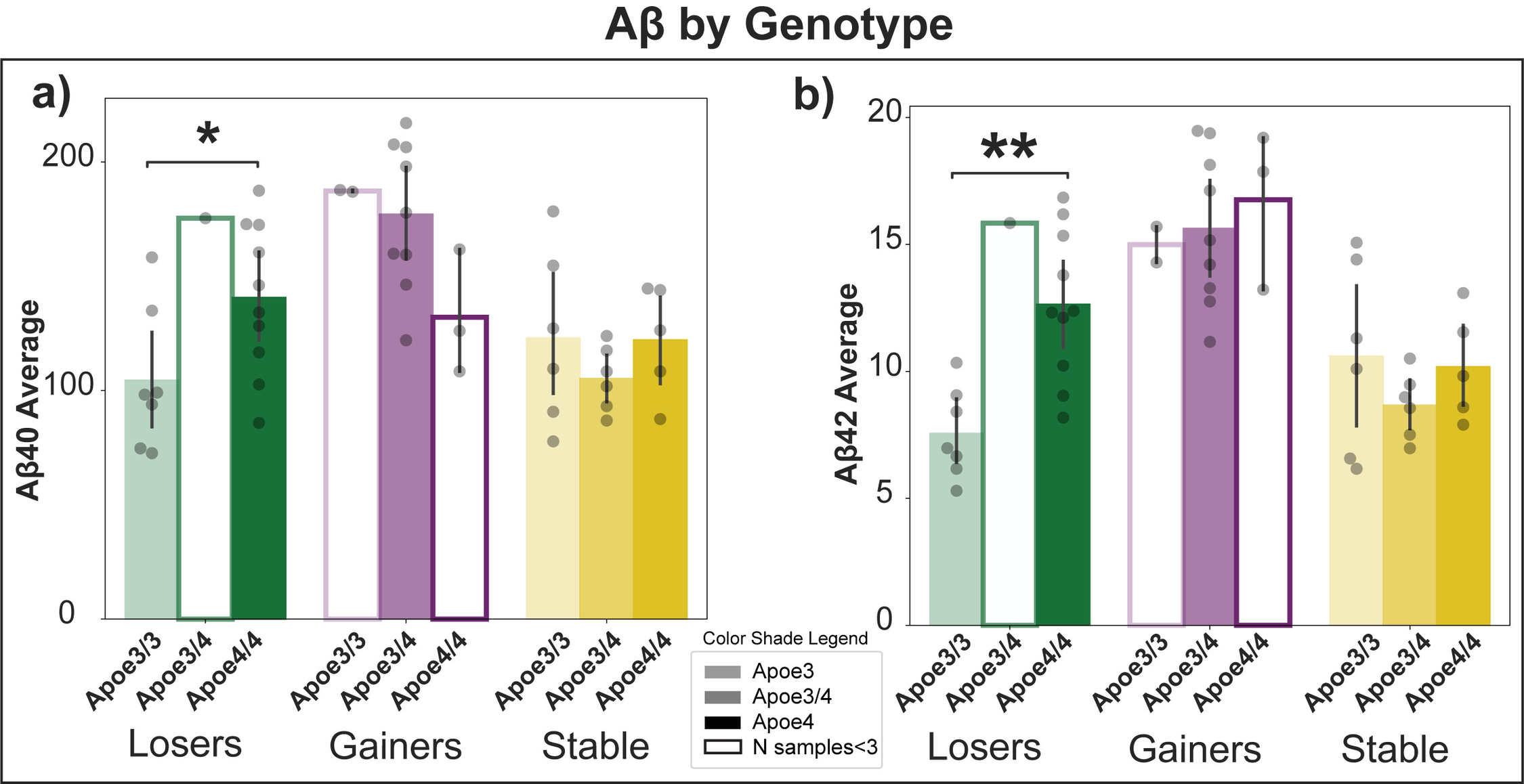

Supplement: S5 Fig — Panels a and b show average levels of Aβ40 and Aβ42 concentrations, respectively. Bars featuring only the outline color represent groups with fewer than 3 samples. (TIF) [file pone.0314097.s008.tif]

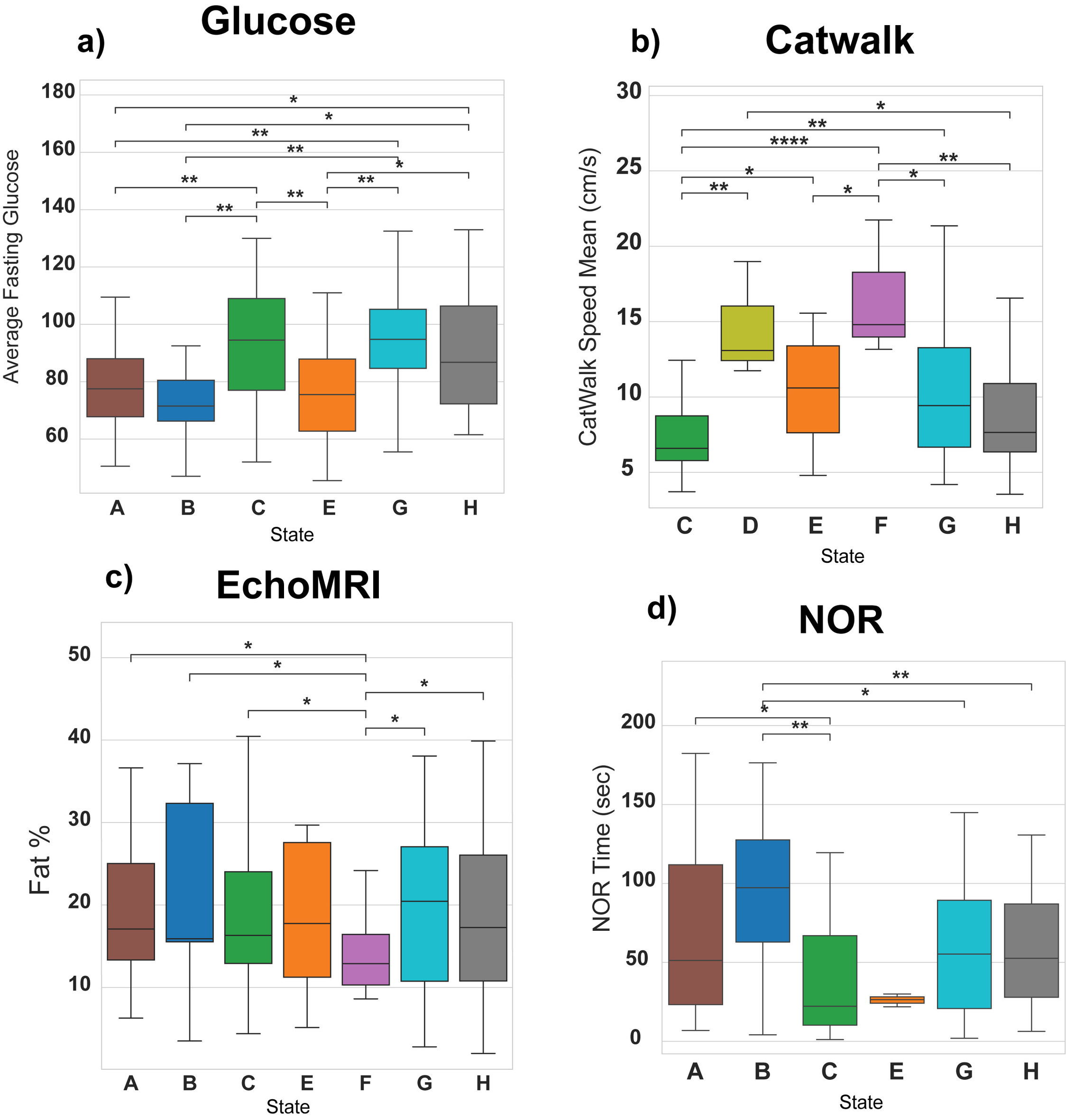

Supplement: S6 Fig — a) shows distribution of age for blood glucose, CatWalk, EchoMRI, and Novel Object Recognition (NOR), while b shows the distribution of mice weights in grams at the time of cross-sectional data collection. These distributions highlight the variability in age and weights of mice for which physiological and behavioral data were available. Panels a) to d) show blood glucose, CatWalk, EchoMRI, and NOR across AHMM-inferred hidden states. Statistical differences between data distributions across the estimated trajectories are highlighted with an asterisk. P-value annotation legend: *: 1.00e-02 < p-value < = 5.00e-02; **: 1.00e-03 < p-value < = 1.00e-02; ***: 1.00e-04 < p-value < = 1.00e-03; ****: p-value < = 1.00e-04. The AHMM learned transition probabilities from the longitudinal weight data was utilized to estimate the hidden state (potential trajectory group) by including the same variables used as input (age, sex, APOE genotype, and weight) for the cross-sectional data. Using the AHMM probabilities, each CS measurement was associated with one of the 10 possible hidden states. Glucose blood concentration, EchoMRI, CatWalk, and NOR values were subsequently grouped according to the inferred hidden ending state. To determine if significant differences existed between hidden states, we conducted t-tests on the data distributions. (TIF) [file pone.0314097.s009.tif]

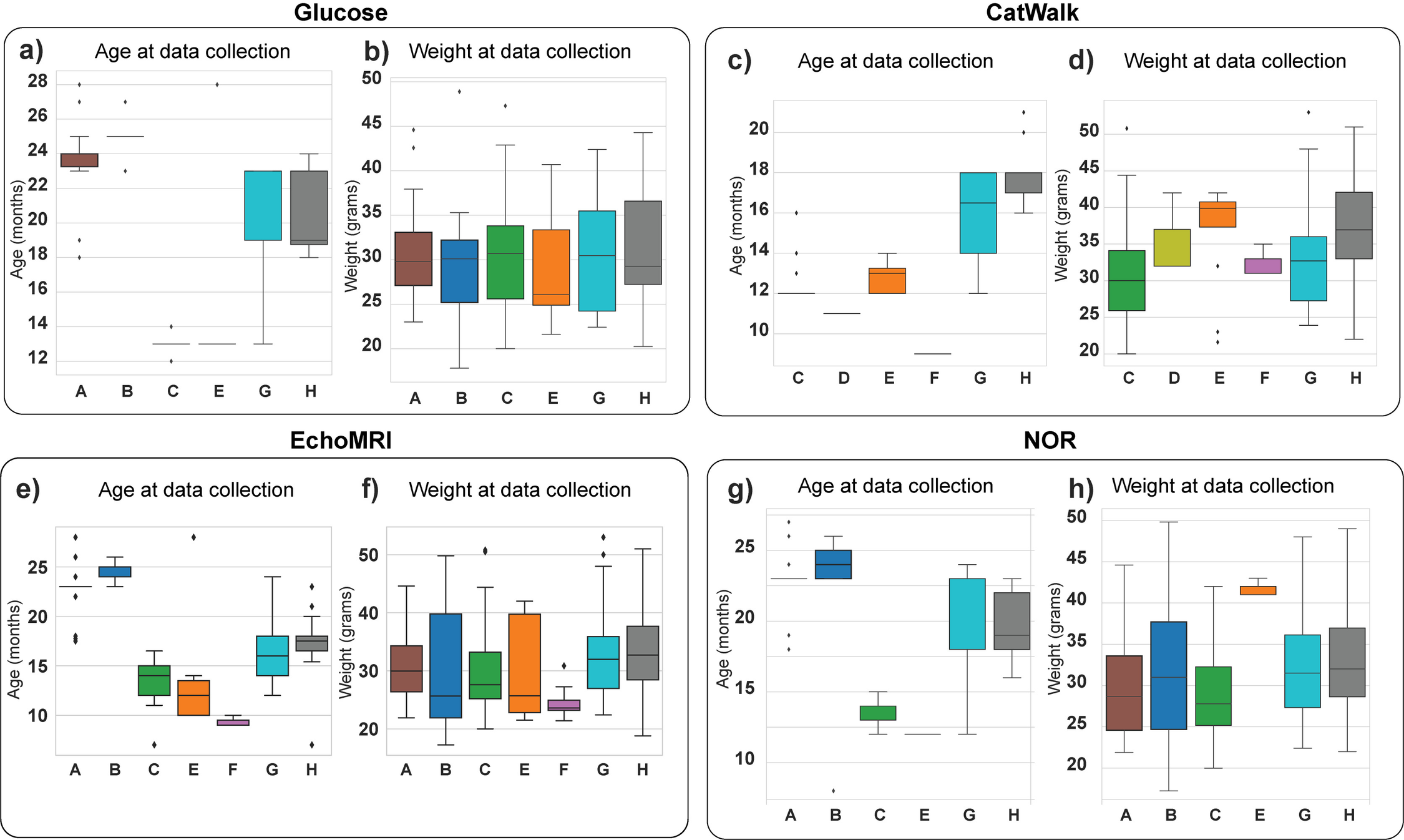

Supplement: S7 Fig — Panel a-j show for each inferred hidden state distribution of the age at the data collection, weight at the data collection, and distribution of the data respectively for EchoMRI, CatWalk, Glucose, and NOR. In the boxplots, straight lines denote that mice in the respective trajectories share the same age, while dots represent outliers. (TIF) [file pone.0314097.s010.tif]
