# Supplementary material for: Weight trajectories in aging humanized APOE mice with translational validity to human Alzheimer’s risk population: A retrospective analysis
Source: PLoS One. 2025 Jan 24;20(1):e0314097. doi: 10.1371/journal.pone.0314097 (PMC11760569; doi:10.1371/journal.pone.0314097)
Supplement: S1 File — (DOCX) [file pone.0314097.s011.docx]

**Supplementary File S1 - Cross-sectional data collection**

**Novel Object Recognition (NOR)**

**Apparatus**

The video recordings were analyzed using ANY-maze software by experimenters blinded to animal sex or genotype (Stoelting Co., Wood Dale, IL; Version 7.0). The software allows location-tracking of animal head and body position, and all analyses were monitored to ensure the software correctly tracked animal head and body position. Areas of interest are defined in the software, including the center zone (central 50% area of the apparatus; 16cm x 13.3cm) and zones drawn around the objects (2 cm away from each side of the object). Movement speed was defined as total distance traveled divided by time spent mobile. This allowed both overall movement analyses as well as numerous object interaction metrics, such as time spent behavior within defined zones around the Novel and Familiar Objects. The variable *Novel Object Time (sec)* corresponds to the total time spent within the designated zone around the Novel Object that was summed for each animal as a measure of performance in the Novel Object Recognition assay (1).

**Procedure**

The workflow, proposed by Leger (1), was utilized for this study. Prior to the commencement of experiments on each day, the animals acclimated to the experimental room for 1 hour. All assays were performed during the same time of day (9am ± 1 hour) to control for any time-of-day effects. On Day 1, the animals were placed in separate boxes and allowed to explore the open field for 15 minutes. On Day 2, the boxes were populated with two identical objects, placed on the opposite edges of the center zone, 10.4 cm from the closest corner. The animals were placed in the center of the box and allowed to explore these objects for 5 minutes. On Day 3, one of the objects was replaced with an identically sized object with a different shape and color (novel object). The object locations were the same as Day 2 and which site became the novel object location was balanced across cohorts to minimize potential location preferences. The animals were again placed in the center of the box and allowed to explore the objects for 10 minutes. Fecal boli counts were measured at the end of each animal’s session as a metric for anxiety level within the environment.

**Video recording analysis**

The video recordings were analyzed using ANY-maze software by experimenters blinded to animal sex or genotype (Stoelting Co., Wood Dale, IL; Version 7.0). The software allows location-tracking of animal head and body position, and all analyses were monitored to ensure the software correctly tracked animal head and body position. Areas of interest are defined in the software, including the center zone (central 50% area of the apparatus; 16cm x 13.3cm) and zones drawn around the objects (2 cm away from each side of the object). This allowed both overall movement analysis as well as behavior within defined zones and object interaction metrics. Movement speed was defined as total distance traveled divided by time spent mobile. In addition to movement-related parameters and exploration times, preference index (familiarization phase; [object 1 time]/[combined object time]*100%) and discrimination index (DI; recognition phase; [novel object time – familiar object time]/[combined object time]) were calculated as metrics of object preference. Values near 50% (preference index) and 0 (discrimination index) indicate no object preference.

**Fasting blood glucose**

Fasting blood glucose levels were measured via lateral saphenous vein bleed on right hindlimb after a 16hr fast (5pm-9am). Animals were restrained with 50mL conical tube, leg shaved, lanced with a 25-gauge needle, and two glucometer tests performed and averaged with a monthly calibrated glucometer (MyFreeStyle Lite, Abbott Laboratories, Abbott Park, Illinois). Gauze and gentle pressure were applied at the injection site until bleeding stopped. Readings differing by >=10mg/dL necessitated a retest.

**CatWalk**

Gait analysis using the CatWalk method was be performed using the CatWalk™ XT (Noldus Information Technology, Wageningen, The Netherlands). Animals were acclimated to the room for 1 hour, with white noise and lux levels <10. Animals were placed in the corridor (~130 x 68 x 152 cm (~51” x 27” x 60”) with walls adjusted to animal width) of the CatWalk apparatus. A camera, located under the floor, capture real-time footprint images. A run is defined as the animal passing through the field of view of the camera at a steady, uninterrupted pace for no longer than 10 seconds with less than 120% variation in speed; trials consist of ~5 uninterrupted runs.

1. Leger M, Quiedeville A, Bouet V, Haelewyn B, Boulouard M, Schumann-Bard P, et al. Object recognition test in mice. Nature protocols. 2013;8(12):2531-7.
