# Supplementary material for: Weight trajectories in aging humanized APOE mice with translational validity to human Alzheimer’s risk population: A retrospective analysis
Source: PLoS One. 2025 Jan 24;20(1):e0314097. doi: 10.1371/journal.pone.0314097 (PMC11760569; doi:10.1371/journal.pone.0314097)
Supplement: S2 File — (DOCX) [file pone.0314097.s012.docx]

**Supplementary File S2**

**Autoregressive Hidden Markov Models trajectory validation**

To validate the weight trajectories identified by the Autoregressive Hidden Markov Model (AHMM), we used Cross-Sectional (CS) data available for our cohort of weighted mice. Note that these CS data were not included in the learning process of the weight trajectories by the AHMM, ensuring an independent validation approach.

CS data included measurement of glucose blood concentration, EchoMRI, CatWalk, and NOR values. These data were available in a subset of mice and were collected at different ages (Fig S1) as described in File S1.

We employed the AHMM along with standard statistical analyses to assess potential significant differences in measured variables across the inferred hidden states identified by the AHMM.

For each CS measurement, weight, age, sex, and APOE genotype at the time of data collection were provided as inputs and set as evidence in AHMM. In instances where weight data was not exactly available at the same time as CS data collection, we allowed for a window of 1 month for alignment. Subsequently, based on the AHMM probabilities, one of the 10 possible hidden states was estimate for each CS data point.

Glucose blood concentration, EchoMRI, CatWalk, and NOR values were subsequently grouped according to the related inferred hidden ending state (Fig S6). The related weights and age at collection of the data are reported I Fig S7. Note that CS measurements were not included as inputs to the AHMM, but they were used to determine if significant differences of CS values existed between hidden states. To this end, we conducted t-tests on the data distributions. The identification of statistically significant differences across hidden states in the cross-sectional data validates the AHMM's ability to identify trajectories that differ not only in weight but also in other independent data not utilized during the model's training process.
